# Spice-Derived Bioactive Compounds Confer Colorectal Cancer Prevention via Modulation of Gut Microbiota

**DOI:** 10.3390/cancers14225682

**Published:** 2022-11-19

**Authors:** Marco Dacrema, Arif Ali, Hammad Ullah, Ayesha Khan, Alessandro Di Minno, Jianbo Xiao, Alice Maria Costa Martins, Maria Daglia

**Affiliations:** 1 Department of Pharmacy, University of Napoli Federico II, Via D. Montesano 49, 80131 Naples, Italy; 2 Postgraduate Program in Pharmacology, Federal University of Ceará, Fortaleza 60430372, Brazil; 3 Department of Medicine, Combined Military Hospital Nowshera, Nowshera 24110, Pakistan; 4 CEINGE-Biotecnologie Avanzate, Via Gaetano Salvatore 486, 80145 Naples, Italy; 5 Department of Analytical and Food Chemistry, Faculty of Sciences, Universidade de Vigo, 32004 Ourense, Spain; 6 International Research Center for Food Nutrition and Safety, Jiangsu University, Zhenjiang 212013, China; 7 Department of Clinical and Toxicological Analysis, Federal University of Ceará, Fortaleza 60430372, Brazil

**Keywords:** dietary spices, gut microbiota, colorectal cancer, prevention

## Abstract

**Simple Summary:**

Colorectal cancer (CRC) is a highly prevalent form of cancer, and represents a serious, global, health threat. Available therapeutic approaches have failed to provide control over the increasing prevalence and incidence of CRC. In this context, CRC prevention may provide a fruitful strategy. Edible plants have the potential to alter numerous molecular pathways, which may fight against the pathogenesis of CRC, and the gut microbiota could represent this link between dietary factors and CRC incidence. Spices and their active principles are reported to alter the balance of gut microbial species by increasing eubiotic and decreasing dysbiotic strains. The present study is designed to highlight the cancer prevention potential of spices while focusing mainly on gut microbial modulation. Although several spices and their active components have shown CRC-preventing properties via gut microbial modulation, the literature is still very limited, and expanding the literature going forward is essential before any conclusion can be drawn.

**Abstract:**

Colorectal cancer (CRC) is the second most frequent cause of cancer-related mortality among all types of malignancies. Sedentary lifestyles, obesity, smoking, red and processed meat, low-fiber diets, inflammatory bowel disease, and gut dysbiosis are the most important risk factors associated with CRC pathogenesis. Alterations in gut microbiota are positively correlated with colorectal carcinogenesis, as these can dysregulate the immune response, alter the gut’s metabolic profile, modify the molecular processes in colonocytes, and initiate mutagenesis. Changes in the daily diet, and the addition of plant-based nutraceuticals, have the ability to modulate the composition and functionality of the gut microbiota, maintaining gut homeostasis and regulating host immune and inflammatory responses. Spices are one of the fundamental components of the human diet that are used for their bioactive properties (i.e., antimicrobial, antioxidant, and anti-inflammatory effects) and these exert beneficial effects on health, improving digestion and showing anti-inflammatory, immunomodulatory, and glucose- and cholesterol-lowering activities, as well as possessing properties that affect cognition and mood. The anti-inflammatory and immunomodulatory properties of spices could be useful in the prevention of various types of cancers that affect the digestive system. This review is designed to summarize the reciprocal interactions between dietary spices and the gut microbiota, and highlight the impact of dietary spices and their bioactive compounds on colorectal carcinogenesis by targeting the gut microbiota.

## 1. Introduction

Colorectal cancer (CRC) is the most prevalent form of carcinoma, and represents a leading component of the global health burden. Advancements in treatment methods, colonoscopy, and avoidance of risk factors, such as smoking and red meat consumption, have contributed to a decline in CRC cases over the last three decades in the United States [1,2]. However, similar declines have only been observed in developed countries [3]. Despite innovative strategies of treatment and diagnosis, CRC remains the third most common cancer and the second leading cause of mortality across the globe. In the year 2018 alone, 1.8 million new CRC cases were recorded including 881,000 deaths [4]. CRC cases may rise to 2.5 million by the year 2035 [3]. The modifiable risk factors for CRC include obesity [5], cigarette smoking [6], heavy alcohol use [7], poor diet [8], and a sedentary lifestyle [9]. The genetic contribution towards CRC is in the range of 12–35% as demonstrated in twin and family studies [10,11]. While 60–65% of cases arise sporadically without any family history of CRC [12]. This sizeable sporadic contribution to the instigation of CRC shows the significance of environmental factors, which play a large role in causing CRC [13]. Among environmental factors, infectious agents are responsible for 15 percent of all cancers [14]. Colorectal carcinogenesis is a process involving years of development, possibly taking decades. In such scenarios, early life risk factors and lifestyle modification are pertinent contributors [15]. The current rise of CRC in the young adult population in the US is alarming [2], and this supports the concept that early life risk factors provide a major impact on CRC carcinogenesis [16].

The human microflora counts around thirty trillion bacteria without considering fungi and viruses. The microbiota is not only altered by the environment but also by the relationship between the host and the symbiotic organisms [17]. The total number of microbial cells is 10 times greater than that of human somatic cells [18,19,20,21,22] and these include over 1000 different species of bacteria populating our gut. Most of these belong to the *Firmicutes* and *Bacteroides* phyla and are linked to the protection of the host, as they can produce metabolites and bioproducts promoting a protective effect against different pathologies. The dietary compounds and vitamins produced by these bacteria are considered protective elements against the infiltration of gut pathogens and the development of pathologies [23,24,25]. The impairment of the microbiota could lead to dysbiosis, and several studies sustain this link between tumorigenesis and microbiome diversity, thanks to the combination of next-generation sequencing and computational analysis [26,27,28,29,30,31]. A well-regulated microbiome is essential for maintaining the homeostasis of the metabolism and immune response, in fact, several clinical studies underline how the immunotherapeutic response could be influenced by the gut microbiome, suggesting that treatments could be enhanced or depressed according to the gut microbiota status [31,32,33,34].

Another important role of the microbiome is the recognition of the conserved regions of Gram-negative pathogenic bacteria after the production of immunoglobin G antibodies [35,36]. However, the composition and the alteration of the microbiota are also related to different host life stages and diets [37,38,39,40,41]. It is calculated that 20% of all cancers are related to dysbiosis, and with this perspective, probiotics could be used as therapeutic agents to re-establish the normal microbial environment, enhancing the immune response to counteract tumor growth. Literature data have shown that gut microbiota may provide the missing link between dietary factors and CRC incidence [42]. Some dietary components, such as saturated fats, processed carbohydrates, red meat, and ultra-processed food can affect the gut microbiota and lead to inflammation [43], and inflammation is a known factor for 20–30% of CRC cases and is acknowledged as the principal driver of tumorigenesis [44,45,46].

While chemotherapy and radiotherapy are the key approaches employed for the treatment of patients with cancer, both are associated with serious adverse events that may outweigh their therapeutic benefits in certain cases. Drug resistance is another concern that is very common for anticancer therapies and may result in failure of the treatment [47]. Nature has provided a range of preventive and therapeutic agents with the potential to fight against the most devastating chronic disorders including cancer [48,49]. Edible plants containing phytochemicals are known to alter numerous molecular pathways that may impact anticancer effects (i.e., oxidative stress, inflammatory cascade, apoptosis, epigenetic regulation, p53 signaling pathway, nuclear factor kappa-light-chain-enhancer of activated B cells (NF-ĸB) pathway, mitogen-activated protein kinases (MAPKs), proteasome pathway, insulin-like growth factor-I mediated signal transduction pathway, matrix metalloproteinases (MMPs), vascular endothelial growth factor, Hippo signaling pathway, phosphoinositide 3-kinase–protein kinase B–mammalian target of a rapamycin signaling pathway (PI3K/Akt/mTOR), cyclooxygenase-2, and the Janus kinase–signal transducer and activator of transcription signaling pathway) [50,51,52,53,54,55,56,57]. 

Some spices such as turmeric, black cumin, ginger, ginseng, garlic, saffron, and black pepper, are potential sources of cancer prevention owing to their natural bioactive compounds (curcumin, thymoquinone, piperine, and capsaicin) [58,59,60]. About 80% of the world population is currently relying on phytomedicine for their primary healthcare [61], in fact, these natural products are commonly considered a safer alternative for patients, if compared to systematic chemotherapeutic drugs although their scientific validity and efficacy are currently under analysis [62,63]. These spices and herbs have been used for thousands of years in small amounts thanks to their beneficial effects. In particular, curcuma, ginger, garlic, clove, chili pepper, saffron, and flaxseed seem to inhibit CRC growth thanks to their chemotherapeutic roles [58,64,65,66]. CRC development is sustained by cancer stem cells (CSC), which are self-renewal and pluripotent stem cells able to promote carcinogenesis and the formation of heterogeneous tumors [67]. Increasing evidence sustains the link between microbiota alterations and mature tumor formation. In particular, their metabolome [68] can promote pro or anti-carcinogenic actions. The preservation of the CSC is essential and mediated by several phytochemicals such as curcumin, quercetin, lycopene, cinnamic acid, resveratrol, sibilin, and epigallocatechin-3-gallate [EGCG] [69]. The main pathways involved in the regulation of the CSC phenotype are Hedgehog, Notch, and Wnt/β-catenin [70], which are modulated thanks to the colonic microbiota transformation of phytochemicals. At the same time, these substances can modify the microbiota population. Thus, the diet can change the colonic bacteria and vice versa in a triangular rapport where is involved CRC formation.

The previous similar reviews [71,72] focused their attention on the molecular basis of CRC linking the antioxidant/anti-inflammatory activities of these spices or other diet-derived phytochemicals and CRC pathogenesis. In some cases, recent articles also considered the relationship between the dietary compounds and the gut microbiota-derived metabolites without considering that these two aspects are essential for CRC prevention. In fact, year after year, it is clear how new discoveries on CRC lead to the hypothesis that the anti-inflammatory and antioxidant activities of herbs and spices, normally consumed in the diet, are not the only mechanism of action that intervenes in CRC prevention. The role of microbiota, in fact, seems to be crucial in the modulation of the microbiome and control of the CSC population. The aim of this review is to focus on spice-derived bioactive compounds influencing gut microbiota strains, with special reference to CRC prevention.

## 2. Gut Dysbiosis and Carcinogenesis

The maintenance of healthy gut microbiota during an individual’s lifespan, and any potential loss of diversity, is strictly connected with their diet. The progression of a disease could also involve the long-term depletion of specific groups of bacteria, which could be induced by lifestyle changes and other societal factors [37,38]. Healthy conditions are completely different from those of patients affected by dysbiosis. In the first case, the immune system can easily recognize pathogenic microbes, promoting their consequent elimination [73], most gut bacteria are non-pathogenic, and they offer an important defense role in inhibiting colonization by pathogens. The immune cells (i.e., dendritic cells, macrophages, and phagocytes) are involved in the gut microbiome and are essential for the recognition of pathogenic bacteria [74]. Healthy individuals could suffer either mild or severe issues if bacteria translocate across the epithelial mucosa. Kupffer cells may be involved, after the production of endotoxins and viable or dead bacteria. However, in the case of dysbiosis, the commensal bacteria may also spread into extra-intestinal sites and tissues. Obviously, this event can promote septic shocks, sepsis, organ failure, and death [75] over short-term periods. The dysregulation of the microbiota is associated with various pathologies, and this could be also induced using antibiotics which are known to reduce microbiotal diversity. The state of the art sustains that diabetes types 1 and 2, obesity, arthritis, Crohn’s disease, arthritis, and celiac disease are linked with the deregulation of the microbiotal metabolism and inflammation, which promotes the incidence of these pathologies [75,76,77,78,79,80].

Obviously, the microbiota is strongly involved in the absorption and metabolization of nutrients, thanks to the expression of a great number of genes, which are not expressed in our own organism. The impairment and the downregulation of these processes can promote inflammation, which may also lead to cancer in the longer-term [81,82]. The increased incidence and prevalence of cancer over recent decades are mainly due to a higher exposure to cancer-causing molecules, but also to high-fat diets, which promote dysbiosis and the inflammation process [78]. The microbial alteration could be one of the main factors, which contribute to carcinogenesis [83], in fact, different studies have supported the importance of the relationship between carcinogenesis and lifestyle. The inflammation process remains a driving force in the progression of cancer, promoting its development through the production of inflammatory cytokines [84], with microbial dysbiosis leading to increased concentrations of interleukin (IL)-1, 6, 10, and tumor necrosis factor alpha (TNF-α). The production of IL-10 is essential for the body’s elimination of cancer, in fact it is considered the most effective anti-inflammatory cytokine involved in tumorigenesis [85,86,87]. Wnt signaling is involved with NF-ĸB and MAPKs, which together can lead to an increase in oxidative stress and inhibition of apoptosis [88,89]. Animal and human studies have shown that bacteria such as *Fusobacteria, Alistipes, Porphyromonadaceae, Coriobacteridae, Staphylococcaceae, Akkermansia* species and *Methanobacteriales* are predominantly increased in CRC, while *Lactobacillus, Bifidobacterium, Faecalibacterium species, Treponema, Roseburia,* and *Ruminococcus* are known to reduce [90].

The production of toxins can also influence the tumorigenesis process, with *Helicobacter pylori*, *Escherichia coli,* and *Shigella flexneri,* for example, inducing double-strand DNA cuts causing apoptosis or alteration of the cell cycle [91]. Starting from *E. coli*, colibactin and cytolethal distengin toxins induce genomic instability, promoting breaks in the host’s DNA and tumorigenesis [89]. *S. Flexneri* instead produces cysteine proteases, such as virulence gene A and the inisitol phosphate phosphatase D, with the final response, in this case, being necrosis, with the development of cancer and cell death due to the degradation of the *p53* gene and host damage [92]. *Fusobacterium nucleatum* disrupts the junction of β-catenin through the effector adhesin A (FadA); moreover, it is responsible for the production of virulence factor (Fap2) but in this case, it is through the mediation of blocks of natural killer cells (NK cells) through the binding of the NK inhibitory receptor [92,93,94]. *Bacteroides fragilis* produces a toxin responsible for DNA damage after the production of reactive oxygen species and hydrogen peroxide [95], the same is the case for *Enterococcus fecalis*, which is responsible for the production of extracellular superoxide, able to trigger mutations in host DNA [55]. Finally, *Lactobacillus casei* is responsible for the production of the ferrichrome siderophore, which activates c-Jun N-terminal kinase (JNK) signaling and consequent apoptosis [96].

## 3. Gut Microbial Alteration, Chemotherapy, and Cancer Prevention

Our gut contains trillions of microorganisms interacting with the host, and it is important to underline their essential role in bodily function. Digestion, secretion of metabolites, and the intervention of the immune system as cited above, are strictly related to the microbiota. Bacteria-free mouse models underline how dysbiosis is related to immunoglobulin A, lymphadenitis, and the absence of mucus [97,98]. Cancers very often become resistant to the drugs most used for their treatment [99,100], and unfortunately in 90% of cases, this phenomenon is responsible for the patient’s death [101,102,103]. Obviously, this problem requires attention and time to promote the development of new treatments, and the gut microbiota in particular may also influence the efficacy of antitumor therapies [104].

The negative impact of the absence of a microbiota is becoming clearer year after year, with different studies on mice treated with antibiotics underlining the efficacy of chemotherapy and immunotherapy [105]. Moreover, it is possible that the efficacy of chemotherapy treatments may be heightened under normal conditions, promoting the destruction of cancer through the intervention of T-lymphocytes and myeloid cells. The antibiotic treatments applied in certain mice studies [106] can impair the presence of bacteria and the production of cytokines, however further clinical studies are required to confirm these preliminary findings. The combination of metabolomics and metagenomics underlines the importance of the gut-brain axis [107], which regulates the composition of the gut flora through the production of neuro-hormones and hormones. The case of cyclophosphamide is particularly interesting, a chemotherapeutic drug able to promote the T-cell immune response in the presence of commensal microbiota, which translocates from the spleen to the lymph nodes promoting their anticancer effect [94,108]. It appears that *Bifidobacterium* can enhance dendritic cells, promoting the activation of T CD8-positive cells and enhancing the efficiency of anti-programmed death ligand (PDL-1) therapy [109]. The five-year survival rate was found to have increased by 80% for 1000 sarcoma patients treated with killed microorganism activate (*Serratia* and *Streptococcus*) [110]. The T lymphocytes associated with antigen 4 (CTLA-4) seem to have anticancer effects, promoting the production of CTLA-4 inhibitors. In the absence of CTLA-4, germ-free mice registered a positive response against cancer following an exposure to *Bacteroides* [111] underlying the anticancer effects of these molecules.

Only a few studies to date appear to sustain the relationship between cancer prevention and the microbiota. The production of short-chain fatty acids (SCFAs) by microbiota (i.e., *Propionibacteria* such as *P. freudenreichii*) [112,113,114] has an anti-cancer effect [115], inhibiting the deacetylases of cancer cells. Indeed, a lower concentration of butyrate is registered in cancer patients. The production of SCFAs stimulates the production of IL-18, promoting the healing process in mucosal tissues [116]. Probiotic administration also exhibits interesting effects, as it seems to trigger the immune response with an antitumor effect. Gram-negative bacteria activate TLR4 and T-cells, with *Salmonella enterica,* for example, appearing to be very effective against cervical cancer [117]. Finally, *L. casei* stimulates apoptosis in cancer cells thanks to ferricrome production, through the activation of the JNK signaling pathway [89].

## 4. Spice-Derived Phytochemicals and CRC Prevention by Modulating Gut Bacteria for In Vivo Studies

**Figure 1:**
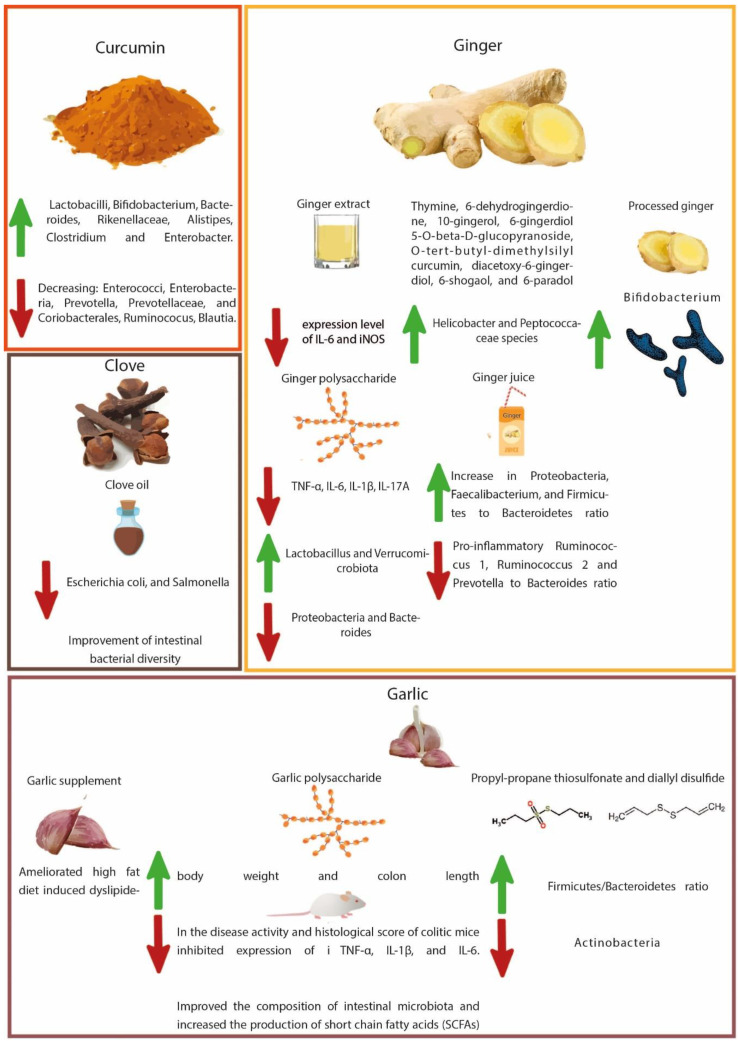
Illustration of gut microbial modulation with curcumin, ginger, garlic, and clove regards to CRC prevention.

**Figure 2:**
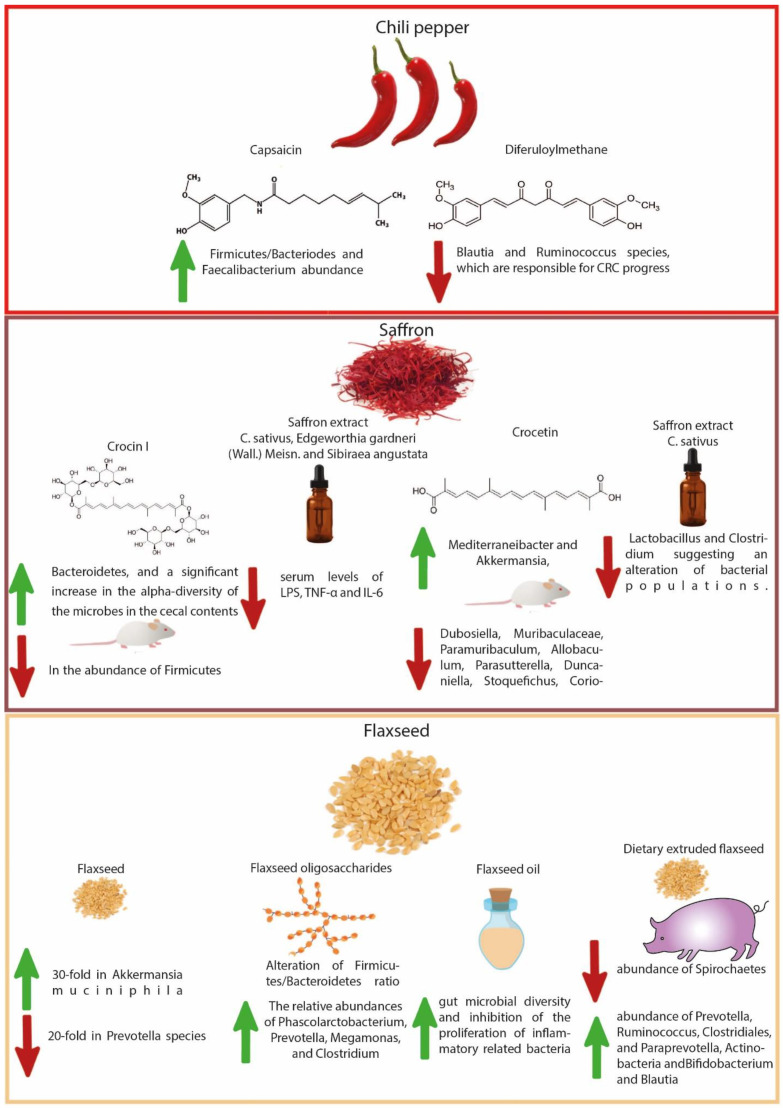
Illustration of gut microbial modulation with chili pepper, saffron, and flaxseed regards to CRC prevention.

Predominantly used as flavoring, coloring, and aromatic agents in beverages and foods, spices are gaining attention for their potential health benefits. The nutritional, antioxidant, anti-inflammatory, antimicrobial, and other medicinal uses of spices have paramount importance [118]. Numerous health benefits of these food adjuncts have been recognized by pioneering experimental studies involving both in vitro and in vivo studies over the past few decades, including their antioxidant and anti-inflammatory potential, digestive stimulant effects, hypolipidemic actions, anti-lithogenic properties, antidiabetic influence, antimutagenic, and anticarcinogenic potentials [119]. Studies have shown that spices and their bioactive compounds may inhibit or even activate pathways related to cell division, proliferation, and detoxification, in addition to immunomodulatory and anti-inflammatory effects [120]. The chemopreventive properties of spice-derived phytochemicals are mainly attributed to the regulation of B-cell leukemia/lymphoma 2 protein, K-ras, MMP pathways, apoptotic pathway, and caspase activation [71]. Considering the scope of the current review, a link between gut microbial modulation by spices and the prevention of CRC pathogenesis has been comprehensively discussed in the sections below. Table 1 summarizes these studies, highlighting the effects of spice-derived phytochemicals on gut microbiota and their ultimate effect on intestinal health. Figure 1 and Figure 2 illustrate the modulation of gut microbes with spices as part of CRC prevention.

### 4.1. Turmeric-Derived Compounds

Curcumin, derived from the roots of the plant known as *Curcuma longa* L., is a natural product that has been extensively studied for the prevention and treatment of cancer [143,144]. Curcumin exerts its anticancer action via various mechanisms, e.g., by inducing apoptosis, thereby inhibiting cell proliferation of cancerous cells, activating caspase, and inducing the expression of anti-oncogenes such as *p53* [145,146]. Interruptions in mucosal barrier function play a significant role in CRC. The persistent inflammation of, and oxidative stress within, intestinal epithelial cells are the most evident causes of colorectal carcinogenesis. Dysfunctions in the mucosal barrier further synergize with this vicious progression of carcinogenesis [147]. The circulating lipopolysaccharide (LPS), due to dysfunction in the gut microbiota, may be a possible cause for the development of chronic inflammatory disorders. The translocation of LPS into systemic circulation occurs due to a dysfunction in the intestinal barrier [148]. The western style diet has been reported to increase intestinal permeability and may be responsible for intestinal barrier dysfunction [149]. Many studies have demonstrated that pretreatment with curcumin attenuates LPS-induced inflammatory cytokines by modulating the p38 MAPK pathway. Curcumin exerts this action most likely on intestinal epithelial cells, thereby reducing intestinal barrier dysfunction [150,151].

The higher concentration of curcumin in the gastrointestinal tract after oral administration suggests that it may regulate the gut microbiota, resulting in various pharmacological actions despite its low systemic bioavailability [121,152]. The present data suggest that curcumin is metabolized by the gut microbiota into different metabolites through diverse pathways, including demethoxylation, hydroxylation, and demethylation. Moreover, these metabolites have been found to be more active compared to the parent molecule curcumin. The higher concentration of curcumin in the gastrointestinal tract after oral administration shows its preferential impact on gut microbiota composition. On the other hand, the processing of the parent molecule transforms it into its bioactive metabolites, resulting in its various therapeutic and pharmacological actions [153]. 

As evidenced in many studies, curcumin shows a direct influence on gut microbiota by increasing the ratio of beneficial bacteria compared to pathological ones [154,155,156]. An in vivo study has shown a significant effect of curcumin on numerous bacterial families in the gut including *Prevotellaceae*, *Rikenellaceae*, and *Bacteroidaceae* [155]. Curcumin administration considerably alters the ratios of beneficial and pathogenic intestinal microflora by enhancing the number and diversity of *Lactobacilli* and *Bifidobacterium*, and decreasing the bacterial load of *Enterococci*, *Enterobacteria*, *Prevotellaceae*, and *Coriobacterales*, thus explaining its immune modulation and anti-tumor effects in the colon [121]. Curcumin administration to mice significantly increased the number of *Bacteroides*, *Rikenellaceae*, *Alistipes*, and *Bacteroidaceae* while decreasing the number of *Prevotella* and *Prevotellaceae* [122]. The number of *Prevotella* has been observed to be higher in patients with CRC, compared to cancer-free patients [157].

In patients with CRC, increased levels of *Ruminococus* species of bacteria have been noticed in the gut microbiota [158,159]. Interestingly, a pilot study elucidated that curcumin, when used as a dietary supplement, reduced *Ruminococus* and *Blautia* bacterial species, and increased the population of *Clostridium* and *Enterobacter* in gut microbiota [123]. The suppressive activity of curcumin on gut microbiotal species shows its anticancer potential in preventing CRC. Through modulating gut microbiota, the administration of nanoparticles of curcumin in mice has demonstrated increased numbers of butyrate-producing bacteria, increased fecal butyrate levels, and suppressed NF-ĸB activation, in colonic epithelial cells. Moreover, it also downregulated the expression of mucosal mRNA in inflammatory mediators [124].

Supplementation of rats with curcumin showed improvements in fecal microbes (reduced *Coriobacterales* and increased *Lactobacillales*), resulting in the regulation of the host immune system, which in turn lowered oxidative and inflammatory stresses, and hyper-immune activation, which may lower the incidence of inflammatory gastrointestinal disorders such as inflammatory bowel disease (IBD) [125]. Literature also reported the eradication of *H. pylori* production with curcumin treatment and its attachment to the human gastric adenocarcinoma cell lines die to its anti-adhesion properties [160,161,162]. Treatment of animals (infected with *Toxoplasma gondii*) with curcumin not only reduced the number of pro-inflammatory *Enterobacteria* and *Enterococci*, but also increased the abundance of anti-inflammatory *Lactobacilli* and *Bifidobacteria* [126]. Oral supplementation of curcumin alleviated acute inflammation of the small intestine by downregulating the Th1-type immune response and preventing bacterial translocation by maintaining the intestinal-barrier function [149]. It inhibited mRNA expression on the mucosa on inflammatory mediators and activated NF-ĸB in colon epithelial cells accompanied by enhanced butyrate-producing bacteria and fecal butyrate levels.

### 4.2. Ginger-Derived Compounds

Ginger rhizome (*Zingiber officinale* Roscoe) belonging to the plant family *Zingiberaceae*, is extensively used as a hot dietary spice in foods and drinks because of its distinctive flavor [163]. Ginger rhizome has a rich chemistry, containing phenolic compounds, terpenes, polysaccharides, organic acids, and raw fibers [164]. The volatile oil components of ginger include sesquiterpenes, zingerberene, curcumene, farnesene, and 40 different monoterpenoid hydrocarbons [165] while the main non-volatile active compounds of ginger include geingerols, shogoals, paradols and zingerone [166,167]. The active constituents [6]-shogaol and [6]-gingerol have shown anti-proliferative activity against various forms of gastrointestinal cancer [167].

As evidenced by numerous studies, ginger extract has a protective activity against ulcerative colitis, a chronic IBD of unknown pathology [168,169,170]. Recently Guo et al. [127] identified the mechanism by which ginger ameliorates dextran sulfate sodium (DSS) induced ulcerative colitis. They found that oral administration of ginger extract modulates the gut microbiota, where it reduces the population of pathogenic bacteria such as *Lactobacillus murinus*, *Lachnospiraceae bacterium 615*, and *Ruminiclostridium_sp. KB18*. Moreover, the ginger extract also reduces the expression level of mRNA of inflammatory cytokines, such as IL-6 and inducible nitric oxide synthase. These studies show that ginger most likely modulates the gut microbiota to reduce inflammation, consequently preventing CRC.

An in vivo study demonstrated a decrease in susceptibility to DSS-induced colitis in mice with ginger extract (containing 16-compounds including thymine, 6-dehydrogingerdione, 10-gingerol, 6-gingerdiol 5-O-β-D-glucopyranoside, O-tert-butyl-dimethylsilyl curcumin, diacetoxy-6-gingerdiol, 6-shogaol, and 6-paradol) following antibiotic exposure in early life [128]. Supplementation to mice with ginger extract for 4-weeks ameliorated weight loss, colon shortening, inflammatory cascade, intestinal barrier dysfunction, and gut dysbiosis. It increased the bacterial diversity and altered the abundance of *Helicobacter* and *Peptococcaceae* species, modulating gut microbial structure and composition adversely affected by antibiotic exposure. A Japanese traditional herbal medicine (Daikenchuto), containing processed ginger, ginseng, and Chinese or Japanese pepper, significantly promoted the growth of *Bifidobacterium adolescentis*, but not that of *E. coli* and *Fusobacterium nucleatum,* in human fecal samples, suggesting an in vitro bifidogenic effect that may contribute to the beneficial effects on colon [129]. Ginger polysaccharides relieved DSS-induced ulcerative colitis in mice via gut microbial modulation, maintaining intestinal barrier integrity [130]. Ginger polysaccharides reduced the level of colonic pro-inflammatory mediators such as TNF-α, IL-6, IL-1β, IL-17A, and interferon (IFN)-γ. In addition, ginger polysaccharides restored gut barrier function, restrained apoptosis, and modulated gut microbiota (by balancing *Firmicutes/Bacteroidetes* ratio, increasing *Lactobacillus* and *Verrucomicrobiota*, and decreasing *Proteobacteria* and *Bacteroides*). An intervention with ginger juice in healthy adults decreased the relative abundance of pro-inflammatory *Ruminococcus_1* and *Ruminococcus_2* and *Prevotella* to *Bacteroides* ratio, with an increase in *Proteobacteria*, *Faecalibacterium*, and *Firmicutes* to *Bacteroidetes* ratio [131].

### 4.3. Garlic-Derived Compounds

Garlic (*Allium sativum* L.), belonging to the plant family *Liliaceae,* is a widespread dietary spice consumed around the globe [171,172]. Garlic consists of various bioactive compounds such as saponins, phenolic compounds, organosulfur compounds, and polysaccharides [173]. The presence of bioactive organosulfur compounds in garlic raises the possibility of anticancer activity [174,175,176,177]. Garlic has a paradoxical effect on the gut microbiota, however, whole garlic supplementation has revealed that it increases the α-diversity of the gut microbiota and as a result ameliorated high-fat diet-induced dyslipidemia [178]. Similarly, the GarGIC Trial results showed that the administration of Kyolic aged garlic extract lowered blood pressure in hypertensive patients by reducing arterial stiffness, inflammation and improving the gut microbiotal profile [179]. The anticancer action of garlic has been explored by its interaction with multiple pathways in carcinogenesis. More experimental and clinical trials are necessary to identify the role of garlic in cancer and particularly in CRC via gut microbiota modulation.

*A. sativum* polysaccharides (200 or 400 mg/kg/day) demonstrated anti-inflammatory activities via modulation of gut microbiota in an experimental model of DSS-induced colitis [132]. Garlic polysaccharides increased body weight and colon length with a decrease in disease activity and histological scores of colitic mice as well as inhibiting the expression of inflammatory mediators i.e., TNF-α, IL-1β, and IL-6. Moreover, they improved the composition of intestinal microbiota and increased the production of SCFAs. The key intestinal microbial strains associated with the inflammatory intestinal conditions identified were *Muribaculaceae*, *Lachnospiraceae* NK4A136 group, *Lachnospiraceae*, *Helicobacter*, *Mucispirillum*, *Ruminococcus 1*, and *Ruminiclostridium 5*. Propyl-propane thiosulfonate (one of the biologically active compounds present in *A. sativum*) modulated immune responses, contributing to anti-inflammatory effects in experimental colitis [133]. The immunomodulatory effects of propyl-propane thiosulfonate were supported by reducing the in vitro production of pro-inflammatory cytokines (TNF-α, IL-1β, and IL-6) and downstream regulation of MAPK-signaling pathways (p44/42 ERK and p38), and in vivo by improving the intestinal epithelial barrier integrity, reducing the expression of pro-inflammatory mediators (TNF-α, IL-1β, IL-8, IL-17, and iNOS), and restoration of gut microbial alteration induced by DSS exposure (increased *Firmicutes/Bacteroidetes* ratio and decreased *Actinobacteria*). On the contrary, another study showed alteration of gut microbiota with diallyl disulfide and induction of fatty liver in the same fashion as caused by a high-fat diet [180].

### 4.4. Clove-Derived Compounds

Clove (*Syzygium aromaticum* (L.) Merr. and L.M.Perry) belongs to the *Myrtaceae* plant family, and is one of the oldest and most valuable dietary spices [181]. The major bioactive constituents of clove oil are eugenol (70–90%) eugenyl acetate, β-caryophyllene, and various sesquiterpenes [182]. Other phytochemicals from clove include bicornin, eugenitin, myricetin, gallic acid, methyl salicylate, methyl amayl ketone, vanillin, ellagic acid, kaempferol, stigmasterol, oleanolic acid, β-caryophyllene and crategolic acid [183]. Considering the broad phytochemistry and biological activities of clove, it has the therapeutic potential to prevent various types of cancers and other diseases [184].

Regarding the effect of clove against CRC, an active fraction of clove extract has demonstrated an anti-proliferative effect against CRC (HCT-116) cells. The active fraction of clove extract induced apoptosis in HCT-116 cell lines, autophagy and inhibited the phosphorylation of the PI3K/Akt/mTOR signaling pathway [185]. In another study, ethyl acetate extract of cloves demonstrated antitumor activity both in in vivo and in vitro models. The clove extract shows dose-dependent induction of apoptosis and has downregulated cell cycle proteins. The authors suggested that clove extract has the potential to be used as a therapeutic herb for treating CRC [186]. Similarly, eugenol has anti-inflammatory effects as observed in mice with DSS-induced colitis, where eugenol treatment has ameliorated the colonic inflammation and oxidative stress in the DSS group [187]. 

An intake of *S. aromaticum* oil 1.5 mL/kg in the diet administered to quails led to an improvement in body weight, activities of antioxidant enzymes, lipid profile, and intestinal bacterial diversity [134]. The coliforms, *E. coli*, and *Salmonella* species were found to be lowered in the ileal contents of quails supplemented with *S. aromaticum* oil, suggesting a reduction in intestinal pathogens, aiming to promote a healthy intestinal status.

### 4.5. Chili Pepper-Derived Compounds

Chili pepper belongs to the *Capsicum* genus, a member of the family Solanaceae. The use of chilies as complementary and alternative medicine in developing countries is rapidly increasing. Alkaloids are the most active compounds present in *Capsicum*, known as capsaicinoids, such as capsaicin, dihydrocapsaicin, nordihydrocapsaicin, norcapsaicin, nornordihydrocapsaicin, homocapsaicin and homodihydrocapsaicin [188]. To date, no clinical investigation has confirmed the effects of capsaicin in human colon cancer, and few studies are focused on the relationship between capsaicin consumption and microbiota alterations [189]. 

A recent study on 512,000 adults revealed that consumption of spices is associated with a lower risk of GI cancer after 5 years of consumption, however, capsaicin also seems to have a negative effect on human health, even if most studies underline that only high doses seem to be harmful. An inverse association was found between spicy food consumption and CRC risk for those who never/rarely consumed and consumed monthly, 1–2 days/week, 3–5 days/week, and 6–7 days/week [190]. This could be related to an increase in butyrogenic bacteria and a decrease in LPS-producing bacteria. Another study found that consumption of 5 mg/d or 10 mg/day capsaicin on a regular basis increased *Firmicutes/Bacteriodes* and *Faecalibacterium* abundance. This event leads to an increase in glucagon-like peptide 1 and gastric inhibitory polypeptide with a decrease in ghrelin [135]. Diferuloylmethane is another interesting compound, which can influence the progress of CRC by changing the gut microbiome. Different studies registered lower intestinal inflammation through a reduction in NF-ĸB in colonic epithelial cells. Another positive effect is the growth of T cells in CD4+ Foxp3+ DSS colitis models and the reduction of *Blautia* and *Ruminococcus* species, which are responsible for CRC progress [191]. 

### 4.6. Saffron-Derived Compounds

Saffron (*Crocus sativus,* L) belongs to the plant family *Iridaceae*, and has been used as a food additive for centuries [192,193]. The phytochemistry of saffron reveals more than 150 compounds, principally comprising flavonoids, apocarotenoids (picrocrocin, crocin, and crocetin), safranal, terpenes, aromatic hydrocarbons, alkaloids, and amino acids [194]. Saffron and its bioactive constituents have the potential to prevent and treat various types of cancer, as evidenced by multiple studies [195]. Crocin significantly prevented DSS and azoxymethane-induced colitis by reducing the level of mRNA expression, inflammatory cytokines, and NF-κB, in colorectal mucosa [196]. Similarly, in another in vivo study, crocin synergized the anti-proliferative action of 5-flurouracil via Wnt/PI3K pathway in CRC mice, associated with colitis [197]. 

In a recent study, saffron extract was administered to CRC cells for anti-proliferation and anti-motility progression by targeting MET transcriptional regulator (MACC1) expression [198]. This accumulating evidence shows the therapeutic potential of saffron in the prevention of CRC via gut microbiota modulation. Crocin-I ameliorated the disruption of gut dysbiosis in mice induced by chronic corticosterone administration. High-throughput sequencing of 16s rRNA demonstrated that crocin-I could mitigate gut dysbiosis through significant decreases in the abundance of *Firmicutes* and an increase of *Bacteroidetes*, and a significant increase in the α-diversity of the microbes in the cecal contents [136]. An herbal formula containing *C. sativus*, *Edgeworthia gardneri* (Wall.) Meisn., and *Sibiraea angustata* modulated gut microbiota with the regulation of gut-liver axis in Zucker diabetic fatty rats [199]. The formula modulated the dysbiosis of gut microbiota and maintained intestinal epithelial homeostasis, resulting in the reduction of serum levels of LPS, TNF-α, and IL-6.

Some pieces of recently published literature have also reported the negative effects of saffron and crocetin on gastrointestinal diseases such as colitis. While investigating the effects of crocetin on the regulation of intestinal barrier function and intestinal microbiota composition in mice, Feng et al. [137] observed prolonged recovery of colitis due to the promotion of inflammation with disturbed intestinal homeostasis under crocetin (10 mg/kg/day for 21-days) with an altered composition of gut microflora and its metabolic products compared to the DSS group. The 16s rDNA sequencing analysis of the feces samples showed a higher abundance of *Mediterraneibacter* and *Akkermansia*, and a lower abundance of *Dubosiella*, *Muribaculaceae*, *Paramuribaculum*, *Allobaculum*, *Parasutterella*, *Duncaniella*, *Stoquefichus*, *Coriobacteriaceae* UCG-002, and *Candidatus*. In addition, crocetin intake also reduced the levels of bile acids including 7-ketodeoxycholic acid, 12-ketodeoxycholic acid, 3-sulfodeoxycholic acid, chenodeoxycholate, 6-ethylchenodeoxycholic acid, glycochenodeoxycholate-7-sulfate, sulfolithocholic acid, and glycocholate in the colon. Another study showed disruption of the cecal microbiome and brush border membrane functionality with *C. sativus* flower water extract [138]. The *C. sativus* extract (1%, 2%, 5%, and 10%) was administered in the amnion of the *Gallus gallus* eggs and was allowed to be consumed by the developing embryo over the next few days. The hatchlings were euthanized, and blood, duodenum, and cecum were harvested for assessment, which showed a significant increase in Mucin 2 gene expression and Paneth cell number proportional to the increase in extract concentration, accompanied by a dose-dependent reduction of *Lactobacillus* and *Clostridium* suggesting an alteration of bacterial populations.

### 4.7. Flaxseed-Derived Compounds

Flaxseed (*Linum usitatissimum* L.) belonging to the Linaceae, is one of the richest dietary sources of omega-3 fatty acids. Other compounds identified in flaxseed include dietary fibers, lignans, and phenolics [200]. Flaxseed is already being extensively used in animal studies to treat cancers of different origins. Numerous studies have demonstrated the prevention of colon carcinogenesis in preclinical studies due to the consumption of flaxseed. Flaxseed possesses immunomodulatory effects, possibly due to prebiotic effects. It maintains the integrity of the intestinal epithelial barrier, inhibiting inflammatory responses and promoting the proliferation of beneficial phyla that may help in preventing CRC development and pathogenesis [201]. Dietary flaxseed supplementation in healthy C57Bl/6 male mice exhibited an alteration in fecal microbial community structure (i.e., a 30-fold decrease in *Akkermansia muciniphila* abundance and a 20-fold increase in *Prevotella* species) along with a significant increase in fecal branched-chain fatty acids, thus decreasing susceptibility to gut-associated diseases including inflammatory pathologies and cancer [139]. 

Flaxseed polysaccharides may reach the colon intact (without being degraded) where changes in carbohydrate contents, reducing sugars, and culture pH suggest that these polysaccharides may be broken down and used by gut microbiota. Zhou et al. [202] observed a modulation of the structure and composition of gut microbiota with flaxseed polysaccharides through the alteration of the *Firmicutes/Bacteroidetes* ratio, and enhanced relative abundances of *Phascolarctobacterium*, *Prevotella*, *Megamonas*, and *Clostridium*, which can degrade polysaccharides. Moreover, the fermentation of flaxseed polysaccharides increased the concentration of SCFAs, particularly propionate and butyrate. Flaxseed oligosaccharides alleviated DSS-induced colitis via the modulation of gut microbiota and repairing of the intestinal barrier in mice [140]. Flaxseed oligosaccharides (200 mg/kg/day) resulted in the improvement of colonic histology, downregulation of oxidative stress markers (malondialdehyde and myeloperoxidase), and suppressed pro-inflammatory cytokines (TNF-α, IL-1β, and IL-6) while increasing the levels of an anti-inflammatory cytokine (IL-10). The 16S rDNA gene high-throughput sequencing indicated an increase in gut microbial diversity and inhibition of the proliferation of inflammatory-related bacteria (*Clostridiales*). An increase in propionic and butyric acids was also observed in mice treated with flaxseed oligosaccharides.

Flaxseed oil supplementation in pigs with intrauterine growth retardation improved intestinal function and immunity (downregulated intestinal expression of MyD88, NF-κB, TNF-α, and IL-10 genes) associated with altered colonic microflora, by decreasing the abundance of *Spirochaetes* and increasing phylum *Actinobacteria*, and genera *Bifidobacterium* and *Blautia* [141]. Treatment of CEABAC10 transgenic mice (with Crohn’s disease) with dietary extruded flaxseed for 12 weeks ameliorated the adherent-invasive *E. coli*-induced intestinal inflammation [142]. Analysis of mucosa-associated microbiota showed a higher abundance of *Prevotella*, *Ruminococcus*, *Clostridiales*, and *Paraprevotella,* in addition to higher butyrate concentration in mice treated with flaxseed. Conversely, ground flaxseeds (rich in omega-3 fatty acids, lignans, and fibers) exacerbated *Citrobacter rodentium*-induced colitis in C57BL/6 mice despite the higher levels of omega-3 fatty acids and cecal SCFAs [203].

## 5. Conclusions

The available literature data suggest that spices and their phytochemicals could be one of the dietary factors that may prevent the risk of CRC development by affecting tumor behavior and targeting numerous molecular mechanisms. Many processes (i.e., oxidative stress, inflammatory cascade, apoptosis, and proliferation) can be influenced by one or more spice-derived phytochemicals. Studies on gut microbial modulation by spice-derived phytochemicals in CRC are still very limited, as spice-derived phytochemicals have been studied in this regard. Thus, the exploration of other spice-derived phytochemicals is essential to provide further insights into the interesting relationship between spice-derived phytochemicals and gut microbiota in CRC. Certain spice-derived phytochemicals have been found to exacerbate gut dysbiosis and intestinal inflammation, such as diallyl disulfide, saffron, crocetin, and ground flaxseeds. So, further confirmation is required on whether this phenomenon will affect the CRC-preventing activity of other spices such as garlic and flaxseed. Additionally, the data reviewed from the literature has mainly been based on preclinical studies, thus robust clinical trials are needed to determine who will benefit from an adequate intake of spice-derived phytochemicals, and what interactions (both positive and negative) may exist among spices with other dietary components or medications (that an individual with CRC may regularly consume). Moreover, the testing of phytochemicals, both in cell cultures and animal studies, at much higher doses than would be regularly ingested, represents pharmacological therapeutic intervention rather than a dietary preventive approach, and thus spice-derived phytochemicals must be tested within the range of dietary doses to assess the actual potential of dietary spices to prevent CRC via gut microbial modulation.

## Figures and Tables

**Table 1:** Summary of anti-colon cancer effects of spice phytocompounds/phytocomplex by modulation of gut microbiota in in vivo studies.

Spice-Derived Compounds	In vivo Study Model	Dose	Treatment Duration	Effect on Gut Microbiota	Comments	References
Curcumin	Mice/Human	100 mg/kg	15 days	↑*Lactobacilli* and *Bifidobacterium*; ↓*Enterococci*, *Enterobacteria*, *Prevotellaceae*, and *Coriobacterales*	May produce immune modulation and anti-tumor effects in the colon	[121]
Curcumin	Mice	NA (meta-analysis)	NA	↑*Bacteroides*, *Rikenellaceae*, *Alistipes*, and *Bacteroidaceae*; ↓*Prevotella* and *Prevotellaceae*	*Prevotella* has been observed as higher in patients with CRC	[122]
Curcumin	Pilot study	1000 mg of curcumin + 1.25 mg black pepper	8 weeks	↓*Ruminococus* and *Blautia*; ↑*Clostridium* and *Enterobacter*	*Ruminococus* species have been observed as higher in patients with CRC	[123]
Curcumin nanoparticles	Mice	0.2 *w*/*w*	7 days	↑number of butyrate-producing bacteria and feal butyrate levels; ↓NF-ĸB activation in colonic epithelial cells	Increased SCFA production may reduce inflammatory processes and intestinal mucosa and promote antitumor effects	[124]
Curcumin	Mice	8 mg/kg/day–162 mg/kg/day	20 days	↓*Coriobacterales*; ↑*Lactobacillales*	Decreased oxidative and inflammatory stresses, and hyper-immune activation	[125]
Curcumin	Mice	20 mg/kg, 100 mg/kg, and 200 mg/kg	10 days	↓*Enterobacteria* and *Enterococci*; ↑*Lactobacilli* and *Bifidobacteria*	Suppressed pro-inflammatory processes and promoted anti-inflammatory effects	[126]
Ginger	Mice	500 mg/kg daily	7 days	↓*Lactobacillus murinus*, *Lachnospiraceae bacterium,* and *Ruminiclostridium* specie KB18	Reduced the expression of mRNA of IL-6 and iNOS	[127]
Ginger	Mice	50 mg/kg	4 weeks	Altered the abundance of *Helicobacter* and *Peptococcaceae* species	Ameliorated weight loss, colon shortening, inflammatory processes, intestinal barrier dysfunction, and gut dysbiosis	[128]
Daikenchuto, Japanese traditional herbal medicine (processed ginger, ginseng, and Chinese or Japanese pepper)	Human colonic microbiota	0.5% wt	48 h	↑*Bifidobacterium adolescentis*	Bifidogenic effects may have beneficial effects on colon	[129]
Ginger polysaccharides	Mice	200 mg/Kg	1,3,5,7 and 9-day dose	Balancing *Firmicutes/Bacteroidetes* ratio; ↑*Lactobacillus* and *Verrucomicrobiota*; ↓*Proteobacteria* and *Bacteroides*	Reduced the level of colonic pro-inflammatory mediators (TNF-α, IL-6, IL-1β, IL-17A, and IFN-γ), restored gut barrier function, and restrained apoptosis	[130]
Ginger juice	Healthy volunteers	500 mg/Kg/day	7 days	↓*Ruminococcus_1* and *Ruminococcus_2* and *Prevotella*/*Bacteroides* ratio; ↑*Proteobacteria*, *Faecalibacterium*, and *Firmicutes*/*Bacteroidetes* ratio	Promoted anti-inflammatory effects in intestinal mucosa	[131]
Garlic polysaccharides	Mice	NA (systematic review)	NA	↑*Bacteroidetes* and *Actinobacteria*; ↓*Firmicutes/Bacteroidetes* ratio	Inhibited the expression of inflammatory mediators (TNF-α, IL-1β, and IL-6); Increased colon length and decrease in the disease activity and histological score of colitis	[132]
Propyl-propane thiosulfonate	Mice	0.01, 0.05, 0.1, 0.5, 1, and 10 mg/kg day	5 days	↑*Firmicutes/Bacteroidetes* ratio; ↓*Actinobacteria*	Improved intestinal epithelial barrier integrity and reduced the expression of pro-inflammatory mediators (TNF-α, IL-1β, IL-8, IL-17, and iNOS)	[133]
Clove oil	Quails	0.75 and 1.5 mL/Kg	42 days	↓*Eescherechia coli*, and *Salmonella* species	Improved body weight, activities of antioxidant enzymes, lipid profile, and intestinal bacterial diversity	[134]
Capsaicin	Healthy adults	10 mg/day	6 weeks	↑*Firmicutes/Bacteriodes* ratio and *Faecalibacterium* abundance	Decreased inflammatory processes and risk factors for CRC	[135]
Crocin-I	Mice	20 mg/kg and 40 mg/kg	3 weeks	↓*Firmicutes*; ↑*Bacteroidetes*	Increased α-diversity of microbes in the cecal contents	[136]
Crocetin	Mice	10 mg/kg	1 week	↑*Mediterraneibacter* and *Akkermansia*; ↓*Dubosiella*, *Muribaculaceae*, *Paramuribaculum*, *Allobaculum*, *Parasutterella*, *Duncaniella*, *Stoquefichus*, *Coriobacteriaceae* UCG-002, and *Candidatus*.	Promoted inflammation with disturbed intestinal homeostasis	[137]
Saffron	Amnion of the *Gallus gallus* eggs	1% CFWE, 2% CFWE, 5% CFWE, 10% CFWE.	Incubation until 21 days	↓*Lactobacillus* and *Clostridium*	Disrupted cecal microbiome and brush border membrane functionality	[138]
Flaxseed	Mice	10% FS diet	1 week	↓*Akkermansia muciniphila*; ↑*Prevotella* species	Decreased susceptibility to gut-associated diseases including inflammatory pathologies and cancer	[139]
Flaxseed oligosaccharides	Mice	50 mg/kg day, 100 mg/kg day, and 200 mg/kg day	14 days	↓*Clostridiales*	Increased colon length, improved colonic histology, decreased oxidative stress markers (malondialdehyde and myeloperoxidase), suppressed pro-inflammatory cytokines (TNF-α, IL-1β, and IL-6), and increased anti-inflammatory cytokine (IL-10); Increased propionic and butyric acids	[140]
Flaxseed oil	Pigs	Flaxseed oil (FO, purity ≥ 98%)	3 weeks	↓*Spirochaetes*; ↑*Actinobacteria*, *Bifidobacterium* and *Blautia*	Decreased intestinal expression of MyD88, NF-κB, TNF-α, and IL-10 genes	[141]
Flaxseed	Mice		12 weeks	↑*Prevotella*, *Ruminococcus*, *Clostridiales*, and *Paraprevotella*	Increased butyrate concentration; Ameliorated the adherent-invasive *E. coli* induced intestinal inflammation	[142]

Colorectal cancer, CRC; short chain fatty acids, SCFAs; nuclear factor kappa-light-chain-enhancer of activated B cells, NF-ĸB; tumor necrosis factor alpha, TNF-α; interleukin-6, IL-6; interleukin-1β, IL-1β; interleukin-17A, IL-17A; interferon-γ, IFN-γ; inducible nitric oxide synthase, iNOS.

## Abbreviations

CRC	colorectal cancer
CSC	cancer stem cells
CTLA-4	T lymphocytes associated with antigen 4
DSS	dextran sulfate sodium
IBD	inflammatory bowel disease
IL-1	interleukin-1
JNK pathway	c-Jun N-terminal kinase pathway
LPS	Lipopolysaccharide
MAPKs	mitogen activated protein kinases
MMPs	matrix metalloproteinases
NF-ĸB	nuclear factor kappa-light-chain-enhancer of activated B cells
NK cells	natural killer cells
SCFAs	short chain fatty acids
TNF-α	tumor necrosis factor alpha
